# Overexpression or Silencing of FOXO3a Affects Proliferation of Endothelial Progenitor Cells and Expression of Cell Cycle Regulatory Proteins

**DOI:** 10.1371/journal.pone.0101703

**Published:** 2014-08-05

**Authors:** Tiantian Sang, Qing Cao, Yuqiang Wang, Fang Liu, Shuyan Chen

**Affiliations:** Department of Gerontology, Xinhua Hospital affiliated to Shanghai Jiaotong University School of Medicine, Shanghai, China; University of Nebraska Medical Center, United States of America

## Abstract

Endothelial dysfunction is involved in the pathogenesis of many cardiovascular diseases such as atherosclerosis. Endothelial progenitor cells (EPCs) have been considered to be of great significance in therapeutic angiogenesis. Furthermore, the Forkhead box O (FOXO) transcription factors are known to be important regulators of cell cycle. Therefore, we investigated the effects of changes in FOXO3a activity on cell proliferation and cell cycle regulatory proteins in EPCs. The constructed recombinant adenovirus vectors Ad-TM (triple mutant)-FOXO3a, Ad-shRNA-FOXO3a and the control Ad-GFP were transfected into EPCs derived from human umbilical cord blood. Assessment of transfection efficiency using an inverted fluorescence microscope and flow cytometry indicated a successful transfection. Additionally, the expression of FOXO3a was markedly increased in the Ad-TM-FOXO3a group but was inhibited in the Ad-shRNA-FOXO3a group as seen by western blotting. Overexpression of FOXO3a suppressed EPC proliferation and modulated expression of the cell cycle regulatory proteins including upregulation of the cell cycle inhibitor p27^kip1^ and downregulation of cyclin-dependent kinase 2 (CDK2), cyclin D1 and proliferating cell nuclear antigen (PCNA). In the Ad-shRNA-FOXO3a group, the results were counter-productive. Furthermore, flow cytometry for cell cycle analysis suggested that the active mutant of FOXO3a caused a noticeable increase in G1- and S-phase frequencies, while a decrease was observed after FOXO3a silencing. In conclusion, these data demonstrated that FOXO3a could possibly inhibit EPC proliferation via cell cycle arrest involving upregulation of p27^kip1^ and downregulation of CDK2, cyclin D1 and PCNA.

## Introduction

Vascular endothelial damage and dysfunction are important physiopathologic characteristics of coronary heart disease. Previous studies have suggested that endothelial progenitor cells (EPCs) contribute to postnatal reendothelialization and neovascularization [1]. Circulating EPCs possess the ability to home to the sites of injured blood vessels or ischemic tissue and differentiate into mature endothelial cells, thereby maintaining endothelial integrity [2]. EPC therapy may promote the development of regenerative medicine and lead to a new therapeutic strategy for cardiovascular diseases (CVD). In fact, several recent studies have been performed on autologous transplantation of EPCs [3]. Patients with coronary artery disease exhibit low levels and dysfunction of circulating EPCs [4]. Also, cardiovascular risk factors and aging have been deemed to result in reduced numbers and impaired functions of EPCs [5]. Therefore, the key factors for cell therapy are proliferative ability and functional status of EPCs.

Recently, Forkhead box type O (FOXO) transcription factors have been a focus of several researchers. Many studies have demonstrated that FOXO factors negatively regulate cell proliferation in various mammalian cell types like glioma cells, vascular smooth muscle cells and endothelial cells [6]. Additionally, we have found that oxidative stress enhances the expression of Forkhead box O3a (FOXO3a) but not FOXO1 and FOXO4 in EPCs [7]. FOXO3a is a member of FOXO transcription factors. Upon dephosphorylation by Akt, FOXO3a is activated and imports into the nucleus from cytoplasm, thereby preventing cell proliferation. The antiproliferation caused by FOXO3a expression is related to accumulation of cyclin dependent kinase inhibitor p27^kip1^ at the protein level, which inhibits cyclin/CDK complexes that are crucial for transition into S phase, followed by cell cycle arrest at G0/G1 phase [8].

The survival and proliferation of EPCs are influenced by many factors. Atorvastatin inhibits EPC senescence and induces EPC proliferation *in vitro* via the regulation of various cell cycle proteins including PCNA, p21 and p27 [9]. Zhu, et al. [10] have shown that homocysteine (Hcy) lowers the proliferative capacity of EPCs through telomerase inactivation. In addition, hemin-induced reactive oxygen species (ROS) promotes EPC proliferation by activating the Akt and ERK signaling pathways [11]. However, the effect of FOXO3a on cell cycle arrest in EPCs has been mentioned in a few studies so far and the mechanisms have not yet been elucidated. In this study, we have investigated the mechanisms governing EPC proliferation and modulation of cell cycle regulatory proteins following FOXO3a overexpression and silencing, respectively.

## Materials and Methods

This work was approved by Medical Ethics Committee Affiliated Xinhua Hospital of School of medicine Shanghai Jiaotong University (Approval No. XHEC-D-2014-003). Written informed consent from the donor of the human umbilical cord blood was obtained.

### Isolation and culture of EPCs

EPCs were isolated from human umbilical cord blood and cultured *in vitro* based on recently published protocols, with a few minor modifications [12]. Briefly, mononuclear cells (MNCs) were isolated by density gradient centrifugation with Histopaque-1077 (Sigma) from human umbilical cord blood according to the manufacturer's protocol. After purification with washing steps, isolated MNCs were plated on six-well culture plates pre-coated with fibronectin (Sigma) and maintained in endothelial cell basal medium-2 (EBM-2) (Clonetics) supplemented with EGM-2 MV SingleQuots (Clonetics) consisting of 5% fetal bovine serum (FBS), hydrocortisone, human fibroblast growth factor-β, vascular endothelial growth factor, insulin-like growth factor-1, ascorbic acid, human epidermal growth factor and GA-1000 (Gentamicin, Amphotericin-B). Cells were cultured at 37°C in a humidified 5% CO_2_ atmosphere. Fresh medium was added three days after non-adherent cells were removed by washing with phosphate buffered saline (PBS). The medium was subsequently replaced every three days. Cell colonies were selected and passaged 14 days after culture. Fluorescence-activated cell sorting (FACS) was operated to purify EPCs. Cells were stained with monoclonal antibodies specific for the following surface antigens: CD34, CD31, and CD133. After incubation at 4°C for 30 min they were analyzed using two-color flow cytometry. The third or fourth passage cells were used for further analysis. The uptake of fluorescence Dil-labeled acetylated LDL (Dil-ac-LDL) was evaluated by HMC confocal microscopy. The binding of UEA-1 can be determined very rapidly using FITC-conjugated UEA-1 (Sigma).

### Adenovirus transfection

Three recombinant adenovirus vectors Ad-TM (triple mutant)-FOXO3a, Ad-shRNA-FOXO3a and Ad-GFP were constructed and preserved in our laboratory. Akt cannot phosphorylate the triple-mutant Ad-TM-FOXO3a, in which the three conserved Akt phosphorylation sites, Thr-32, Ser-253 and Ser-315 were replaced by alanine residues [13]. For transfection, 3×10^5^ EPCs were seeded in triplicate and incubated until approximately 75% confluency in six-well culture plates. EPCs were washed with PBS before transfection with the three recombinant adenoviral vectors (at multiplicity of infection of 40) for 2 hours. After transfection, the cells were washed and incubated with fresh medium for 48 hours. Transfected EPCs were then collected for further analysis.

### Assessment of transient transfection efficiency

Transfection efficiency was assessed by cell counting with microscopy and flow cytometry, and by western blotting to analyze expression of FOXO3a protein (described below). Transfected EPCs cultured in six-well plates were observed under an inverted fluorescence microscope and the number of transfected cells was counted in four random representative fields (at 200× magnification). Additionally, transfected cells were trypsinized with 0.25% trypsin, washed with PBS and resuspended in EBM-2 supplemented with EGM-2 MV SingleQuots (1×10^6^ cells/mL). Untransfected EPCs were similarly harvested as a negative control. Individual samples were transferred into flow cytometry tubes and transfection efficiency analysis for GFP-positive cells was evaluated using a flow cytometer. Dead cells were excluded from the analysis.

### Cell viability assay of EPCs

EPC proliferative activity was assessed using a Cell Counting Kit-8 (CCK-8) (Dojindo). Briefly, equal numbers of EPCs (5×10^3^ cells/well) were seeded in a 96-well plate with 100µL of EBM-2 supplemented with EGM-2 MV SingleQuots. Adherent EPCs were transfected with the three recombinant adenovirus vectors Ad-TM-FOXO3a, Ad-shRNA-FOXO3a and Ad-GFP in sextuplicate. 24, 48 and 72 hours after transfection, 10µL of CCK-8 solution was added to each well of the plate according to the manufacturer's instructions and the plate was incubated at 37°C for 4 hours in a humidified 5% CO_2_ atmosphere. The cell proliferative capability was then determined by measuring the light absorbance at 450nm with a microplate reader.

### Cell growth assay of EPCs

1.5×10^4^ EPCs was seeded in each well of six 6-well plates with 2 mL of EBM-2 supplemented with EGM-2 MV SingleQuots. Adherent EPCs were randomly transfected with the three recombinant adenovirus vectors in nonuplicate; meanwhile, untransfected EPCs were seeded as negative controls. 24, 48 and 72 hours after transfection, EPCs in the experimental groups and control group were respectively harvested in triplicate and cell number was counted by using a haemocytometer for a consecutive three days. According to the number of cells, cell growth curve was drawn.

### Cell cycle analysis

Cell cycle assays were performed by flow cytometry using propidium iodide (PI) staining. Briefly, 48 hours after transfection, each group of EPCs (at approximately 5×10^5^) in triplicate was harvested. After washing twice with cold PBS, the cells were resuspended in 70% pre-cooled ethanol and fixed overnight at 4°C. Next, the fixed cells were washed with PBS and incubated with RNase A and PI at 37°C for 30 minutes in the dark. DNA content was measured by fluorescence-activated cell sorting (FACS) using flow cytometry analysis.

### Western blot analysis

Western blot analysis was performed according to the manufacturer's instructions (Pierce). Briefly, transfected EPCs were lysed and harvested in cell lysis buffer with protease and phosphatase inhibitors (Roche). Protein concentration of the samples was determined using the Pierce BCA Protein Assay Kit (Invitrogen) and the samples were then boiled for 10 minutes. Lysates from EPCs containing 50µg of protein were separated by 10% sodium dodecyl sulfate-polyacrylamide gel electrophoresis (SDS-PAGE) and transferred onto polyvinylidene fluoride (PVDF) membrane (Millipore, Billerica, MA). After blocking by incubation in 5% non-fat dry milk for 1 hour at room temperature, the membranes were incubated with 1∶1000 dilution of primary rabbit antibodies against FOXO3a, p27^kip1^ and CDK2, and primary mouse antibodies against PCNA and cyclin D1 (Cell Signaling Technology) overnight at 4°C, followed by incubation with horseradish peroxidase (HRP)-conjugated secondary antibodies (Cell Signaling Technology) at 1∶1000 dilutions for 1 hour. The protein bands were detected by ECL detection reagents (Millipore, Billerica, MA) and quantified with Quantity One System (Bio-Rad). Anti-tubulin antibody (Sigma, MO, USA) was used as a loading control.

### Statistical analysis

All experiments were independently performed at least in triplicate and the quantitative data were shown as the mean±SD. Statistical significance was evaluated with independent samples t test or one-way ANOVA, when appropriate. Results were considered statistically significant when p<0.05. All analyses were performed with SPSS 17.0 software.

## Results

### Cell morphology

**Figure 1:**
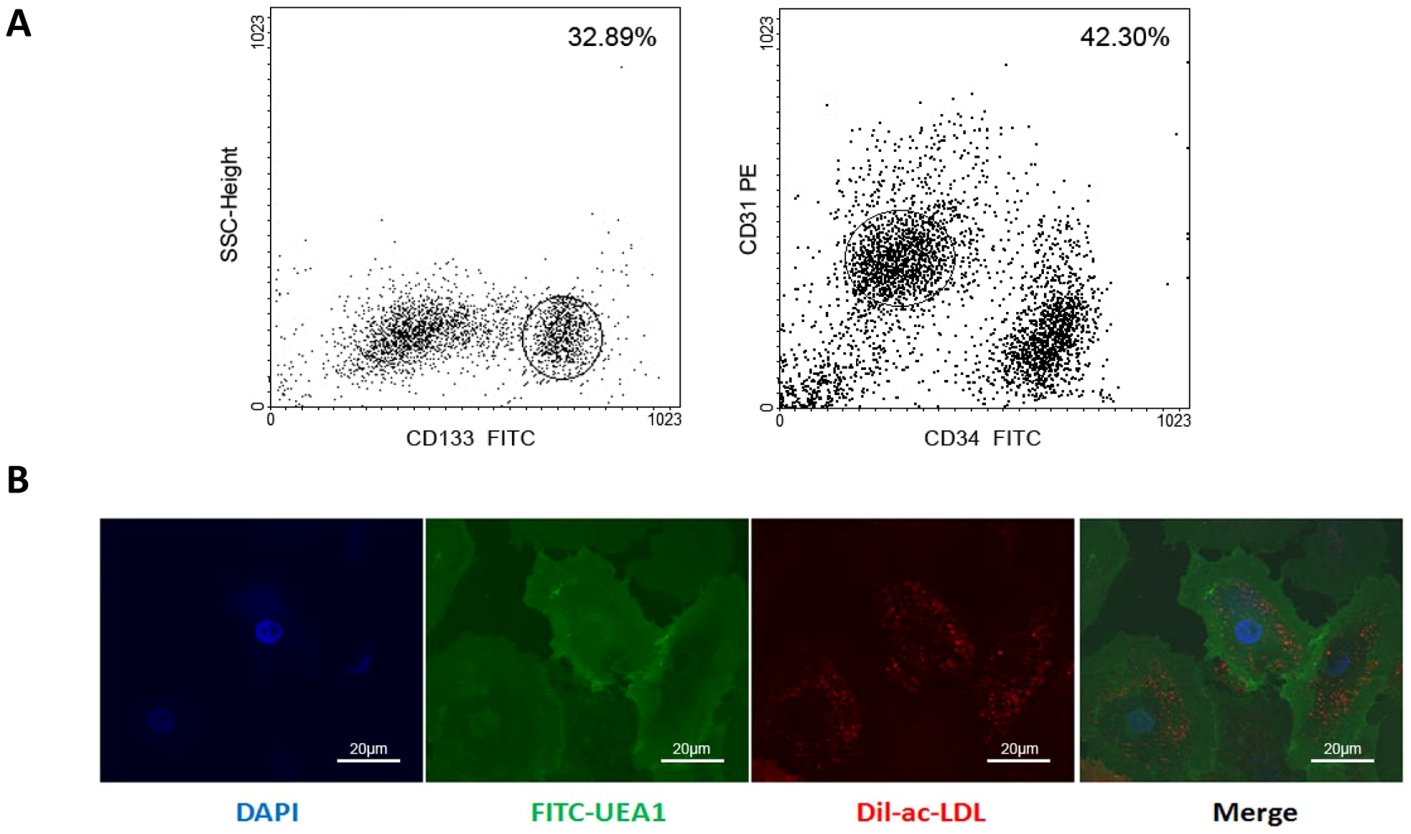
EPCs were isolated and double-labeled for Dil-ac-LDL and UEA1. (A) EPCs(CD133^+^CD34^+^CD31^+^ cells) were isolated using fluorescence-activated cell sorting. (B)Fluorescence microscopy illustrates that endothelial progenitor cells are positive for Dil-ac-LDL(Red) and UEA1(Green) by HMC confocal microscopy.

EPCs were isolated from human umbilical cord blood and morphologically examined with an inverted microscope. After 7 d in culture, some of the MNCs became spindle-shaped and gradually, colony forming units appeared. Later, the colonies became confluent and were selected for passaging. 24 h after passaging, cells expanded rapidly and exhibited cobblestone-shaped monolayer morphology. Consistent with our previous studies [3], EPCs were characterized by flow cytometry, their ability to take up Dil-AcLDL, and to bind lectin-UEA-1 (Fig.1). More than 90% of the cells took up Dil-AcLDL and expressed surface markers such as CD31, Flk-1 and vWF (data not shown).

### The recombinant adenovirus vectors were successfully transfected

**Figure 2:**
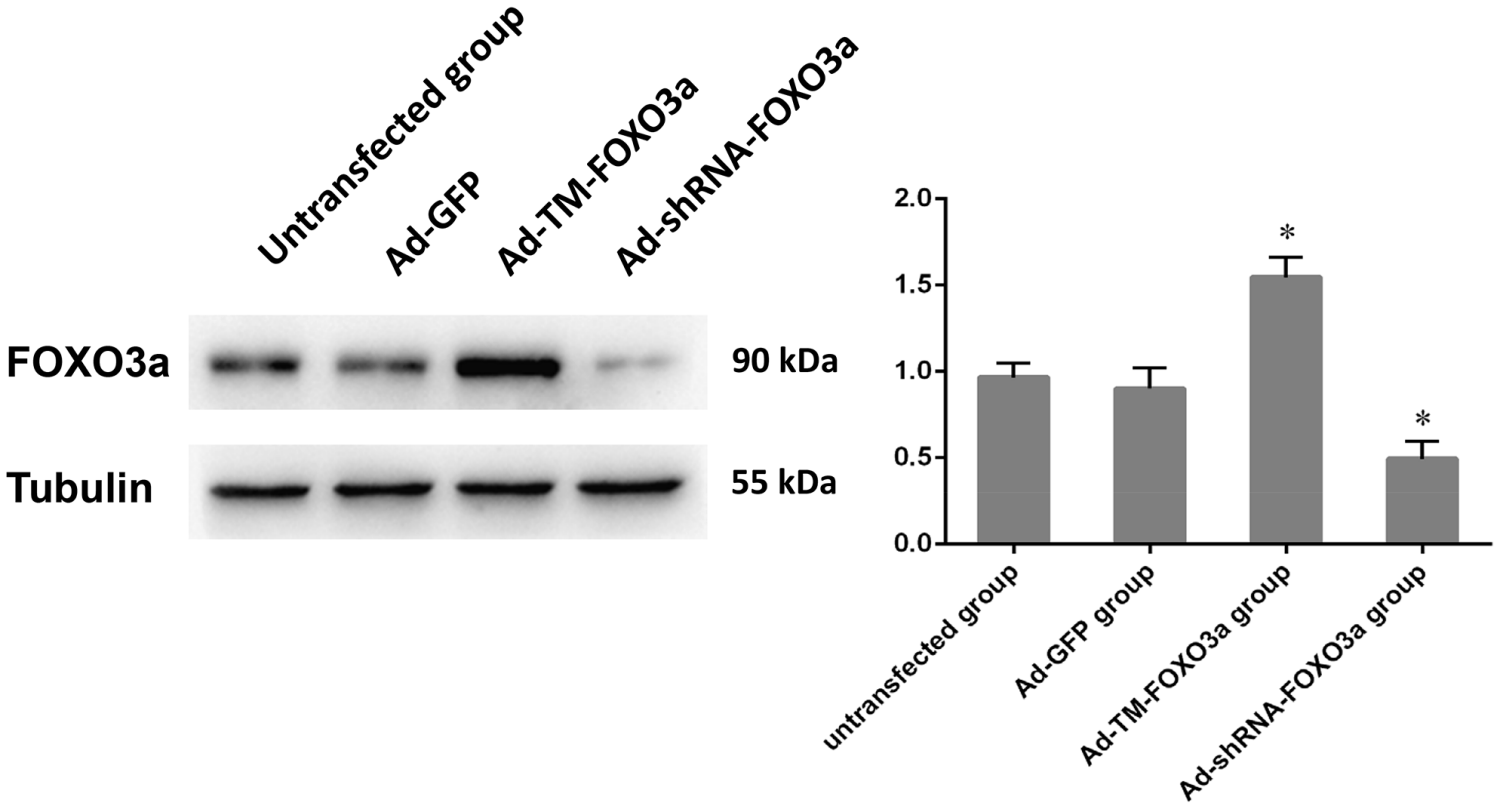
Protein expression of FOXO3a in EPCs transfected with Ad-GFP, Ad-TM-FOXO3a or Ad-shRNA-FOXO3a; meanwhile, untransfected EPCs were operated as a negative control. Blots were scanned, and expression of FoxO3a was quantified by densitometric analysis. The ratios for FoxO3a/Tubulin are shown. Data are mean±SE, n = 3, ^*^p<0.05 vs. Ad-GFP or untransfected group.

To determine if FOXO3a is indispensable for inhibition of EPC proliferation, we had previously constructed two adenoviral vectors Ad-TM-FOXO3a and Ad-shRNA-FOXO3a. The former vector expresses a constitutively active mutant form of FOXO3a, while the latter vector encodes an shRNA that specifically inhibits FOXO3a expression. An adenoviral vector expressing green fluorescence protein (Ad-GFP) was used as a control [7]. In each transfected group, the transfection efficiency measured by GFP expression via fluorescence microscopy was 82.7%, 70.3% and 85.2%, respectively; while the transfection efficiency via flow cytometry analysis was 98.3%, 99.3% and 93.9%, respectively. Also, with respect to transgene FOXO3a protein expression, western blot analysis confirmed that the EPCs were successfully transfected (Fig.2), which provided a strong basis for subsequent experiments. FOXO3a protein exhibited a noticeable increase in the Ad-TM-FOXO3a group as compared to the Ad-GFP group and the untransfected EPCs (p<0.05), whereas the transgene expression in the Ad-shRNA-FOXO3a group was present at fairly low levels as compared to controls (p<0.05).

**Figure 3:**
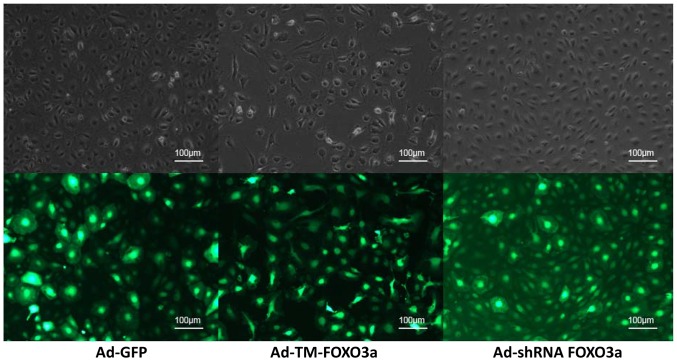
Representative microscopy of the morphology of EPCs at 24-GFP, Ad-TM-FOXO3a or Ad-shRNA-FOXO3a.

Morphology of EPCs varied with changes in FOXO3a activity. After transfection, EPCs were observed under green fluorescent light and ordinary light (Fig.3). Transfected EPCs appeared green by fluorescent microscopy. Following overexpression of FOXO3a, cell division was decelerated and cell number was significantly reduced. Also, the edges of the cells were indistinct and the colonies were not clear. In contrast, cell division was accelerated and cell number was markedly increased when FOXO3a was silenced. Additionally, cell edges were integrated and the colonies were distinguishable. Therefore, these results indicate that FOXO3a affects the growth of EPCs.

### Proliferation of EPCs was impeded by FOXO3a

**Figure 4:**
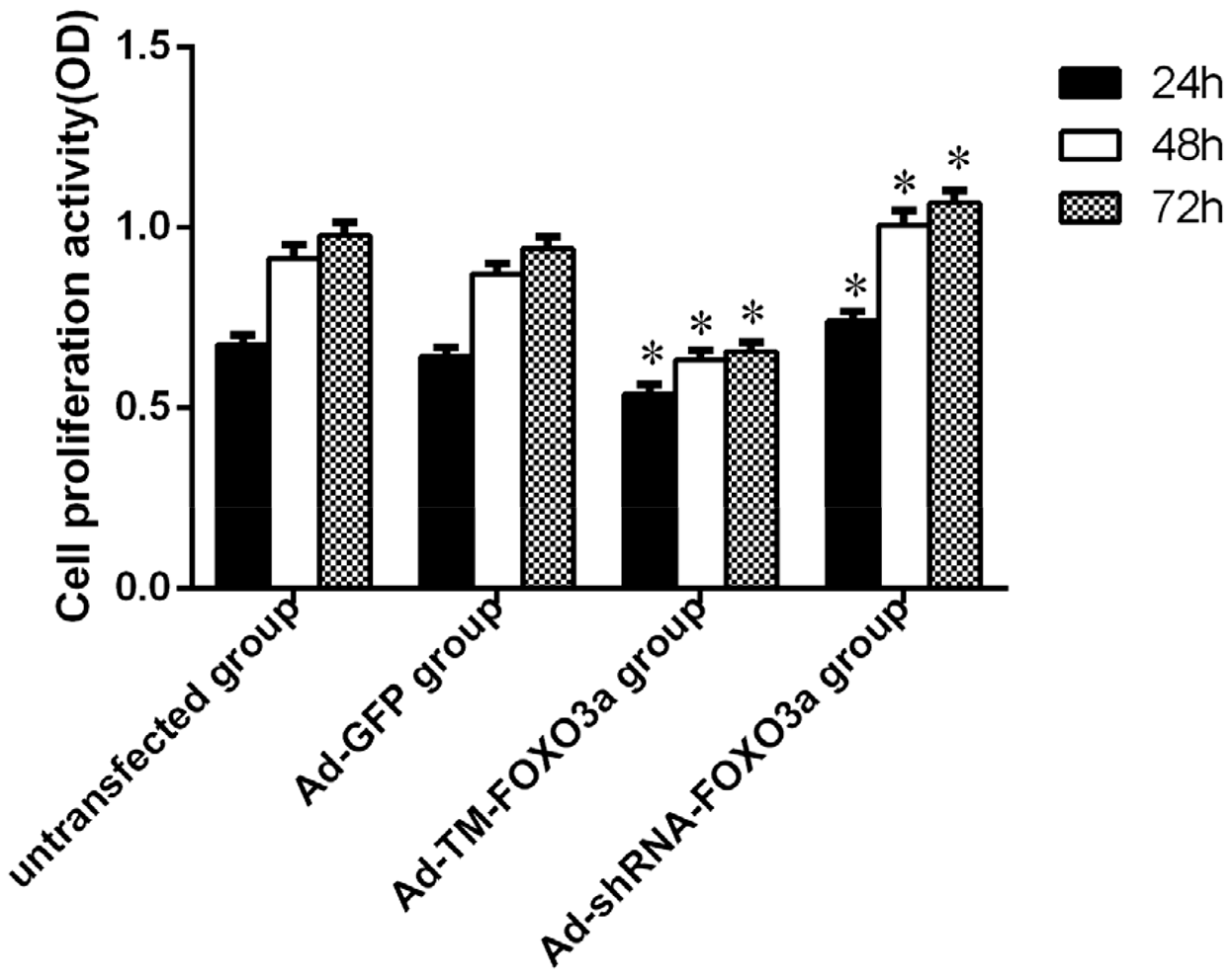
Effects of changes in FOXO3a activity on the proliferation of EPCs via CCK-8 assay. EPCs were transfected with the indicated vectors and cultured in compete EGM-2. Cell viability was determined by CCK-8 assay at the three time points. Data shown are the mean±SE of the ratio for light absorbance at 450 nm. n = 3, *p<0.01 vs. Ad-GFP Group.

To investigate the effects of FOXO3a on EPC proliferation, cell viability was measured at 24, 48 and 72 hours, respectively after transient transfection by CCK-8 assay. As shown in Fig.4, EPC proliferation was strongly attenuated upon transfection with Ad-TM-FOXO3a as compared to the proliferation of EPCs transfected with Ad-GFP at the three time points (0.539±0.064 versus 0.644±0.056 at 24 h, *n* = 6, p<0.01; 0.634±0.064 versus 0.872±0.067 at 48 h, *n* = 6, p<0.01; 0.657±0.062 versus 0.941±0.081 at 72 h, *n* = 6, p<0.01). Additionally, transfection with Ad-shRNA-FOXO3a significantly enhanced the proliferation of EPCs compared to the control at the three time points (0.740±0.069 versus 0.644±0.056 at 24 h, *n* = 6, p<0.05; 1.007±0.096 versus 0.872±0.067 at 48 h, *n* = 6, p<0.01; 1.068±0.083 versus 0.941±0.081 at 72 h, *n* = 6, p<0.05). In accordance with morphological characteristics of transfected EPCs in each group, the results from CCK-8 assay indicated that FOXO3a hindered EPC proliferation *in vitro*. Furthermore, in each group, the mean absorbance ascended more and more slowly in a time-dependent manner due to cell division till there was no significant difference between 48 h and 72 h (*n* = 6, p>0.05), perhaps due to contact inhibition, and the effect of recombinant adenovirus vectors.

**Figure 5:**
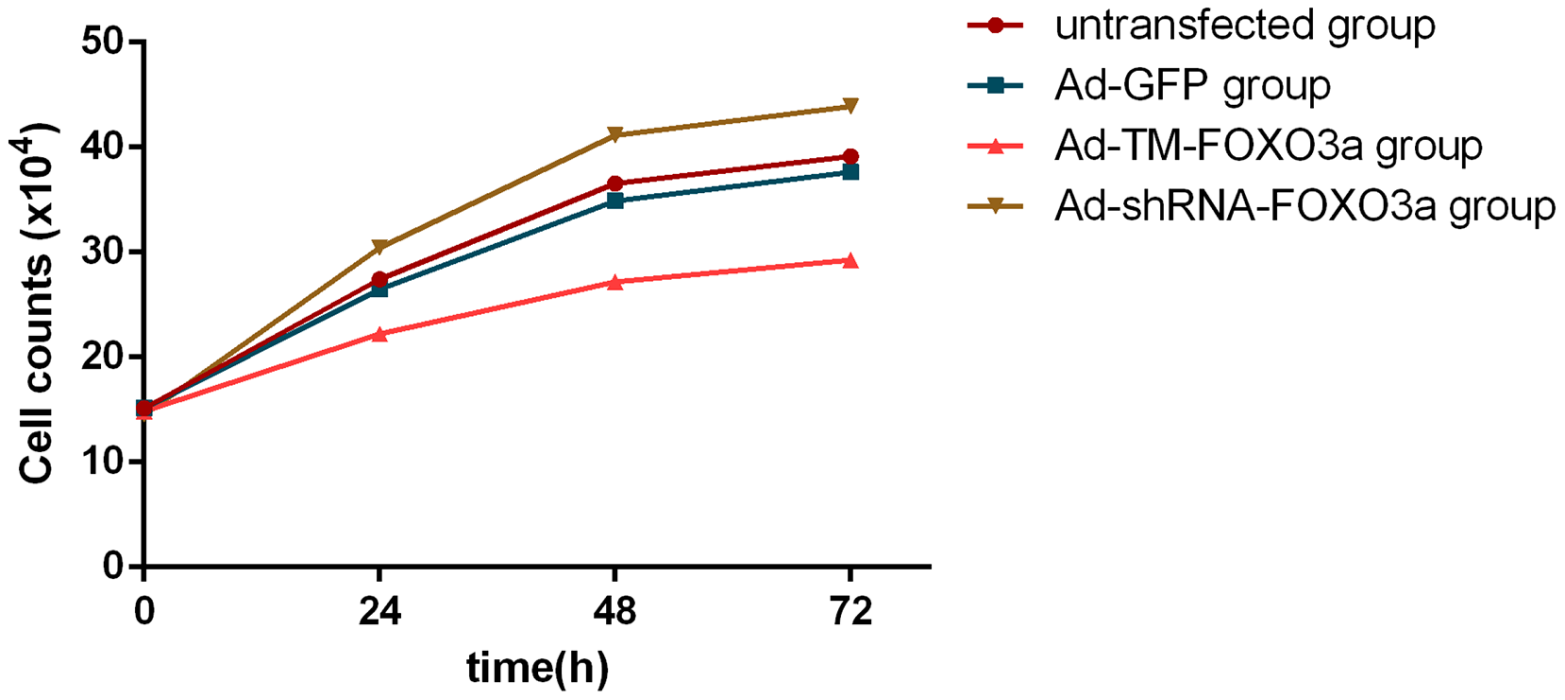
Growth curve of EPCs within 72 hours after transfection. 1.5x10^5^ EPCs were seeded in a 6-well plate and transfected with different recombinant adenovirus vectors. Cell number was counted by using a haemocytometer for a consecutive three days. Growth curve of EPCs showed cell growth pattern within 72 hours after transfection.

According to the growth curve (Fig.5), there were no significant differences in cell growth pattern between untransfected EPCs and EPCs transfected with Ad-GFP. The growth rate in the both groups was faster significantly than that in the Ad-TM-FOXO3a group but slower significantly than that in the Ad-shRNA-FOXO3a group. The growth curve can directly illustrate the EPCs growth pattern, which is consistent with the result in CCK-8 assay.

### FOXO3a led to cell cycle arrest of EPCs *in vitro*

**Figure 6:**
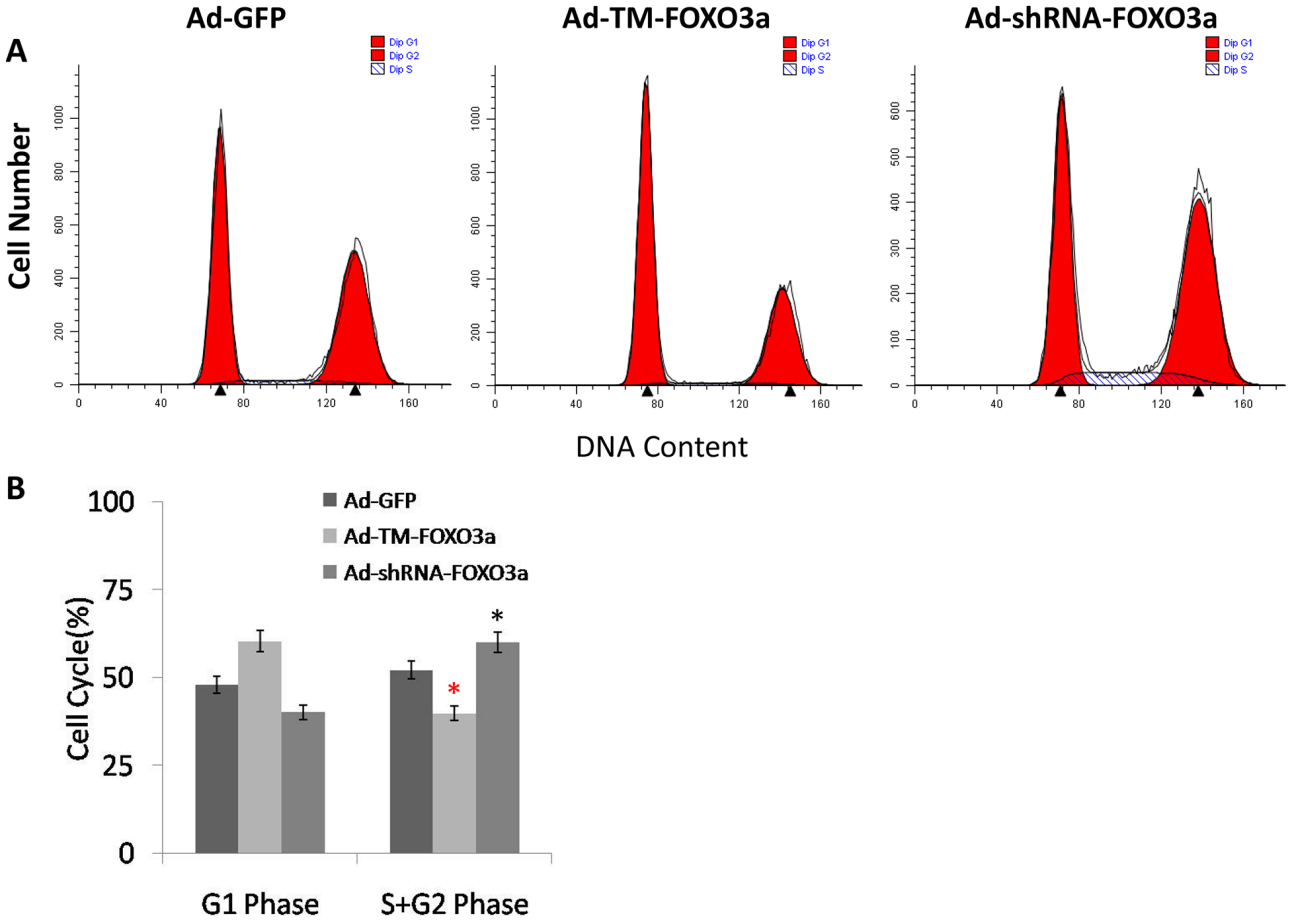
Cell cycle analysis using flow cytometry. (A) The representative FACS plots displayed differences in cell cycle phases of EPCs transfected with Ad-GFP, Ad-TM-FOXO3a or Ad-shRNA-FOXO3a. (B) Cell cycle analysis demonstrated that the proliferation index (the percentage of cells in S- and G2- phases) was significantly elevated after FOXO3a silencing when compared to the proliferation index in Ad-GFP group. Data were presented as the mean±SD of three independent experiments. *p<0.05 vs. Ad-GFP.

Given that FOXO3a retarded the growth of EPCs, we then asked whether these changes occur due to a block at a certain checkpoint of the cell cycle. Hence, EPCs were transfected with Ad-TM-FOXO3a, Ad-shRNA-FOXO3a and Ad-GFP and cell cycle distribution analysis was performed using propidium iodide (PI) staining followed by flow cytometry (Fig.6A). As expected, these data (Fig.6B) showed that in EPCs transfected with Ad-TM-FOXO3a, cell cycle arrest in G1-phase was evidently observed. Conversely, it was confirmed that the proliferation index (the percentage of cells in S- and G2- phases) was significantly elevated after FOXO3a silencing when compared to the proliferation index in Ad-GFP-transfected EPCs, which suggested that cell cycle progression was being triggered. Taken together, these results demonstrate that FOXO3a, which is related to PI3K/Akt cell signaling pathway, impeded cell cycle progression from the G1 phase to the S phase in EPCs, and thereby inhibited EPC proliferation.

### FOXO3a modulated the expression of cell cycle regulatory proteins in EPCs

To further explore the underlying mechanism of how increased FOXO3a activity induced G1 cell cycle arrest in EPCs, the changes in expression of cell cycle regulatory proteins were detected by western blot analysis after transfection. We found that in the Ad-TM-FOXO3a group, expression of PCNA protein, which is associated with DNA synthesis, was noticeably reduced in comparison to the control GFP group. In contrast, increased expression of PCNA was seen in EPCs transfected with Ad-shRNA-FOXO3a as compared to the control. Thus, we deduced that FOXO3a could inhibit cell proliferation, which is correlated with the level of PCNA protein.

**Figure 7:**
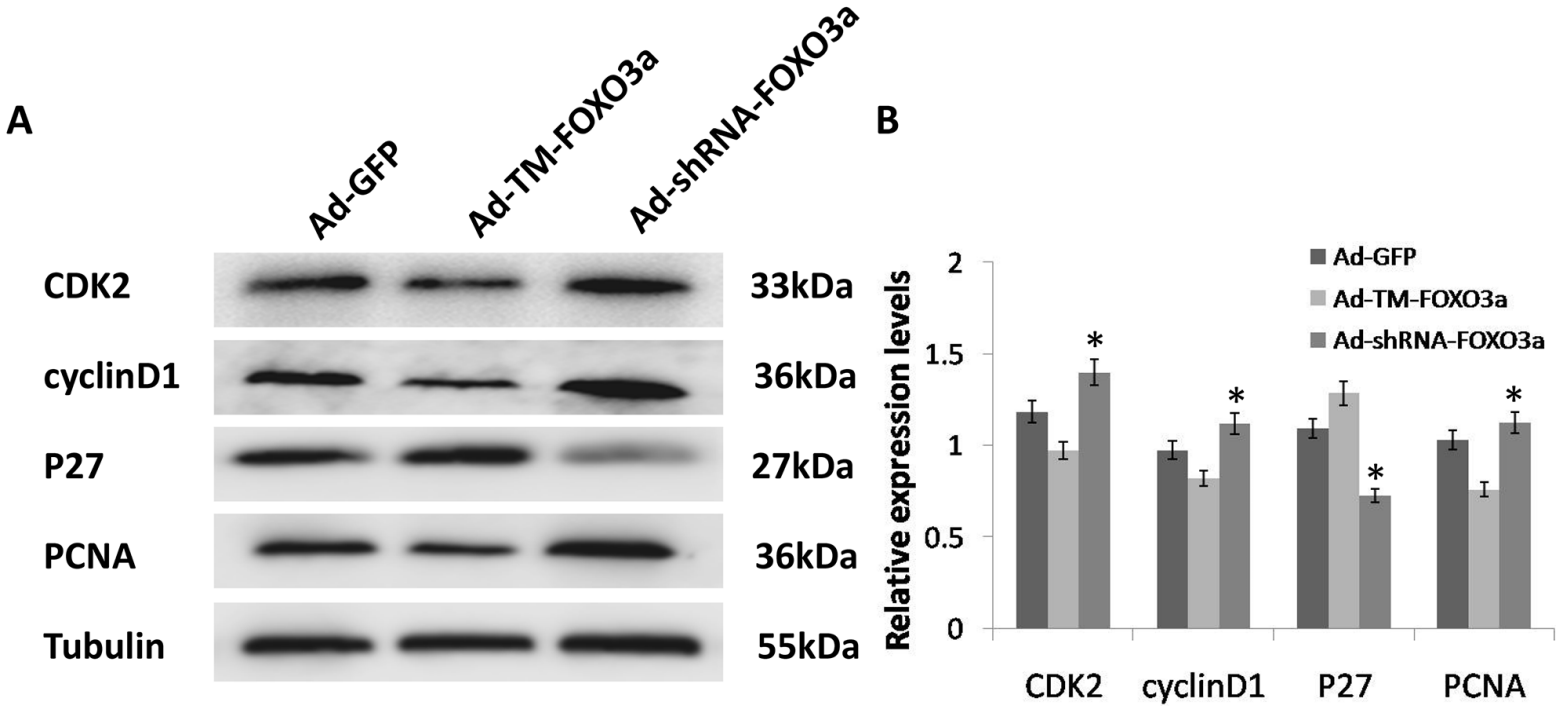
FOXO3a modulated the expression of cell cycle regulatory proteins in EPCs. (A) EPCs were transfected with the indicated vectors. Cells were lysed and protein expression was determined by Western blotting. (B)Blots were scanned, and expression of protein was quantified by densitometric analysis. The ratios of CDK2, cyclinD1, P27 or PCNA to Tubulin are shown. Band intensity analysis was performed with Quantity One Software. Knockdown FOXO3a led to dramatic protein expression increases in CDK2, cyclinD1 and PCNA. Data are mean±SE, n = 3, *p<0.05 vs. Ad-GFP.

The negative cell cycle regulator p27^kip1^ is a cyclin dependent kinase inhibitor (CDKI) that regulates the G1- to S- phase transition [14]. Our results revealed that the level of p27^kip1^ protein was upregulated with decreased cell proliferation as a result of FOXO3a overexpression, whereas it was downregulated upon silencing of FOXO3a, which indicated that FOXO3a expression led to expression of p27^kip1^ protein in EPCs (Fig.7).

Apart from CDKIs, cyclin dependent kinases (CDKs) and cyclins are also important components of the cell cycle clock. In the cell cycle progression from G1- to S- phase, cyclin D1/CDK4, cyclin D1/CDK6 and cyclin E/CDK2 complexes are known to be suppressed by CDKIs such as p27^kip1^ [15]. Changes in cyclin D1 and CDK2 protein levels were evaluated by western blotting. The levels of cyclin D1 and CDK2 were significantly downregulated in EPCs transfected with Ad-TM-FOXO3a, whereas silencing of FOXO3a caused an increase in cyclin D1 and CDK2 expression. Based on the above results, we concluded that FOXO3a might impede EPC proliferation by regulating a series of cell cycle regulatory proteins that control the G1- to S- phase transition.

## Discussion

In the past several years, a considerable amount of evidence has indicated that EPCs play an indispensable role in the regeneration of blood vessels [3]. Meanwhile, much effort has been gradually initiated on exploring various factors and mechanisms, which affect EPC proliferation and functions [16], [17]. A major goal of the present study was to confirm our hypothesis that FOXO3a, a Forkhead box transcription factor, generates antiproliferative effects on EPCs. Our results revealed that when FOXO3a activation is altered, EPCs exhibited alteration in proliferative activity, which is important for blood vessel growth. Moreover, these effects were achieved through regulation of cell cycle distribution and cell cycle regulatory proteins.

In mammals, the FOXO subfamily possesses four members, FOXO1, FOXO3, FOXO4 and FOXO6 (localized in neural cells), and plays an important role in boosting oxidative stress and impeding cell cycle progression and proliferation through diverse post-translational modifications (PTMs) such as phosphorylation, glycosylation, acetylation and ubiquitination [18]. Recently, it has been reported that FOXO3a participates in negative regulation of cellular proliferation in several cell lines and triggers autophagic cell death under persistent stress conditions [19]. Protein kinase B has the ability of promoting cell survival partly by phosphorylating and inactivating FOXO3a in various cell systems [20]. In human umbilical vein endothelial cells (HUVEC), cytochrome P450-derived epoxyeicosatrienoic acids (EETs) stimulated cell proliferation through phosphatidylinositol 3-kinase/Akt-dependent phosphorylation, inactivation of FOXO3a and inhibiting expression of p27^Kip1^ [21].

In the present study on EPCs, we used previously constructed recombinant adenovirus vectors Ad-TM (triple mutant)-FOXO3a, Ad-shRNA-FOXO3a and a control Ad-GFP, among which the triple mutant form, Ad-TM-FOXO3a cannot be phosphorylated by Akt as a result of substitution of the three efficient phosphorylation sites Thr32, Ser253 and Ser315 on FOXO3a with alanine residues (T32A, S253A and S315A, respectively) [13]. After transfection, morphological changes, CCK-8 analysis and growth curve suggested that FOXO3a induced impairment of proliferation of human umbilical cord blood derived EPCs. These results are in accordance with previous reports that showed that in patients with scleroderma, decrease in circulating EPC levels was due to the activation of Akt-FOXO3a-Bim pathway [22]. The negative effects of FOXO3a on EPCs were probably ascribed to nuclear localization of excessively activated FOXO3a [23] and the activation of downstream targets such as Bim and p27^kip1^, resulting in cell cycle arrest. In our previous study, we showed that FOXO3a induced apoptosis of EPCs and impairment of tube formation via upregulation of Bim [7]. We also demonstrated that the changes in the expression level of p27^kip1^ protein, a cyclin dependent kinase inhibitor (CDKI), occurred in parallel with the changes of FOXO3a activity. As shown by flow cytometry, the antiproliferative effect of FOXO3a overexpression was associated with a significant accumulation of G1phase EPCs, and decrease in the proliferative index. This indicated that FOXO3a hampers cell cycle progression, which is in accordance with a prior report that AFX blocks cell cycle progression at G1 phase, dependent on p27 [24].

Both cyclin E/CDK2 and cyclin A/CDK2 are crucial complexes for transition from G1 into S phase, initiation of DNA synthesis and progression through S phase. Zhang S, et al. showed that cyclin E and CDK2 expression levels were negatively correlated with FOXO3a and p27^kip1^ expression in astrocytes following SCI [25]. With respect to cyclin D1, in combination with CDK4/6, it can phosphorylate and inactivate the tumor suppressor Rb, allowing entry into S phase [26]. Recent data showed that cyclin D1-CDK2 complexes are capable of phosphorylating many different substrates in addition to Rb [27]. Moreover, activation of p27^kip1^-cyclin D1/E-CDK2 pathway is involved in polysaccharide (P1)-induced S phase cell cycle arrest in HT-29 cells [28]. The functions of PCNA in cell cycle control have been previously established. PCNA is well known as a characteristic marker of cell proliferation and is involved in DNA synthesis, DNA repair and cell cycle regulation. These functions are achieved by interactions with cyclins and CDKs such as CDK2-cyclin A complexes and cyclin D1 [29]. Similarly, our western blot results demonstrated that the expression levels of cell cycle regulatory proteins CDK2, cyclin D1 and PCNA, which are very important for cell cycle phase transition, were noticeably reduced in EPCs transfected with Ad-TM-FOXO3a, thereby causing cell cycle arrest.

In summary, our results demonstrate that FOXO3a leads to morphological changes and impairment of proliferation in EPCs. In terms of the molecular mechanisms, the subversive effect of FOXO3a is mediated through the regulation of cell cycle related proteins p27^kip1^, CDK2, cyclin D1 and PCNA. Our results support the notion that FOXO3a could serve as a target for EPC therapy in cardiovascular diseases.
